# 1′-Acetoxychavicol acetate promotes caspase 3-activated glioblastoma cell death by overcoming enhanced cytokine expression

**DOI:** 10.3892/ol.2013.1292

**Published:** 2013-04-05

**Authors:** MUSA WILLIAMS, ILLYA TIETZEL, QUINCY A. QUICK

**Affiliations:** Department of Biology, Southern University at New Orleans, New Orleans, LA 70126, USA

**Keywords:** glioblastomas, acetoxychavichol acetate, caspase 3, cytokines

## Abstract

The brain consumes ∼20% of the oxygen utilized in the human body, meaning that brain tumors are vulnerable to paradoxical physiological effects from free radical generation. In the present study, 1′-acetoxychavicol acetate (ACA), a naturally derived antioxidant that inhibits xanthine oxidase, was evaluated for its role as an anti-tumorigenic agent in glioblastomas. The study revealed that ACA inhibited glioblastoma cell proliferation as a consequence of promoting apoptotic cell death by enhancing caspase 3 activity. It was also shown that ACA impaired the migratory ability of glioblastoma cells by decreasing their adhesive properties. Additionally, ACA increased the protein expression levels of the pro-survival signaling cytokines, IL-6 and IL-1α, established cell protectors and survival molecules in brain tumors. Together, these results demonstrate that, despite enhanced expression of compensatory signaling molecules that contribute to tumor cell survival, ACA is an effective pro-apoptotic inducing agent in glioblastomas.

## Introduction

The homeostatic balance of reactive oxygen species (ROS) and redox reactions is critically significant for the maintenance of cellular physiological processes. However, an imbalance of the cellular oxygen environment leads to oxidative cellular stress resulting in deleterious consequences, including DNA damage and the activation of pro-survival signaling molecules (i.e., NFκB and MAPK) that contribute to the etiology and progression of human cancers (1–3). The adverse effects of unregulated ROS levels on human cancers have recently gained considerable attention as therapeutic targets for the treatment of this disease (4–6). Thus, the present study examined the anti-tumor properties of the antioxidant 1′-acetoxychavicol acetate (ACA), a ginger-derived natural product extract from the rhizomes and seeds of *Alpinia galanga,* on brain tumors.

Mechanistically, ACA acts as an inhibitor of xanthine oxidase, which plays a significant role in the catabolism of purines, and catalyzes the oxidation of hypoxanthine to xanthine, and xanthine to uric acid. Additionally, xanthine oxidase plays a significant role in the generation of several ROS, including H_2_O_2_, O_2_^−^ and OH^−^(7). Byproducts of xanthine oxidase oxidation have been implicated in several abnormal physiological processes, including brain ischemia, vascular injury and inflammatory diseases (7). Of particular significance for the present study is the expression of xanthine oxidase expressed at increased levels in brain tumors compared to normal brain tissue (8). In contrast, several scavenging antioxidant enzymes have been shown to be decreased in meningiomas, astrocytomas and medulloblastomas (9). Furthermore, although early studies by Ohnishi *et al*(10) and Tanaka *et al*(11) demonstrated that the xanthine oxidase inhibitor, ACA, was a chemopreventive agent that suppressed tumor formation in the oral cavity and colon of rats, subsequent studies revealed that ACA exerted positive anti-tumorigenic effects on leukemia and breast and multiple myeloma cancers (12–14). The present study demonstrates that ACA antagonizes glioblastoma cell migration and proliferation as a consequence of caspase 3-activated cell death.

## Materials and methods

### Cells conditions and reagents

U373, U87 and A172 glioblastoma cells were purchased from the American Type Culture Collection (Manassas, VA, USA). The study was approved by the Ethics Committee of the Southern University at New Orleans (New Orleans, LA, USA). All cell lines were maintained in Dulbecco’s modified Eagle’s medium (DMEM) (Invitrogen, Carlsbad, CA, USA) containing 10% fetal bovine serum (Invitrogen), 2 mM L-glutamine (Invitrogen), 100 nM minimal essential medium (MEM) non-essential amino acids (Invitrogen), penicillin (5000 units/ml) and streptomycin (5000 *μ*g/ml) (Invitrogen) at 37°C in 5% CO_2_.

### Crystal violet cell proliferation assay

The cells were plated in 12-well plates, treated with 10, 5 and 2 *μ*M ACA and allowed to incubate for 48 h (vehicle controls were treated with dimethyl sulfoxide; DMSO). Next, the tissue culture medium was removed; the cell monolayer was fixed with 100% methanol for 5 min and stained with 0.5% crystal violet in 25% methanol for 10 min. The cells were then washed with distilled water 3 times for 5 min each to remove any excess dye and allowed to dry overnight at room temperature. The incorporated dye was then solubilized in 0.1 M sodium citrate (Sigma-Aldrich, St. Louis, MO, USA) in 50% ethanol. Next, 100 *μ*l of the treated and control samples were transferred to 96-well plates and the optical densities were read at 540 nm using an X-mark microplate absorbance spectrophotometer (BioRad, Hercules, CA, USA).

### Clonogenic survival

The cells were plated for 24 h, treated with 1 *μ*M ACA or DMSO (vehicle) and allowed to incubate at 37°C for 10–14 days. At the termination of the incubation period, the cells were fixed with absolute methanol, stained with 1% crystal violet for 10 min, rinsed in tap water and allowed to dry. Colonies, consisting of ≥50 cells, were then counted to determine the survival fraction.

### Cell motility

Cell motility assays were conducted using polycarbonate membrane inserts of CytoSelect Cell Migration Assays according to the manufacturer’s instructions (Cell Biolabs Inc., San Diego, CA, USA). A cell suspension containing 0.5–1.0×10^6^ cells/ml was prepared in serum-free media with vehicle (DMSO) or 5 *μ*M ACA, while 500 *μ*l of media containing 10% fetal bovine serum was added to the lower chamber of the migration plate. A total of 300 *μ*l of the cell suspension containing vehicle or 5 *μ*M ACA was then added to the inside of each plate insert and allowed to incubate for 24 h at 37°C in 5% CO_2_. Subsequently, the non-migratory cells were removed from the plate inserts (per the manufacturer’s instructions) and the migratory cells were counterstained according to manufacturer’s instructions (Cell Biolabs Inc.).

### Cell adhesion assay

The cell suspensions containing 0.1–1.0×10^6^ cells/ml in serum-free media with vehicle (DMSO) or 5 *μ*M ACA, were plated onto collagen IV adhesion plates for use in a CytoSelect Cell Adhesion Assay (Cell Biolabs Inc.) for 24 h. Subsequently, the media containing vehicle (DMSO) or 5 *μ*M ACA was removed and the cells were stained and solubilized according to the manufacturer’s instructions (Cell Biolabs Inc.). Optical density measurements (540 nm) were then collected for the cell extracts to quantitate the adhesive properties of the cells treated with vehicle DMSO or 5 *μ*M ACA.

### Caspase 3 activity

The cells were plated in serum-free DMEM for 24 h, treated with 2 *μ*M ACA or vehicle (DMSO) and allowed to incubate for 24 h. The cells were then lysed in CelLytic M Cell Lysis reagent (Sigma-Aldrich) and the protein concentrations were determined using the Bradford method. Subsequently, caspase 3 activity assays were conducted with a CaspACE Assay System according to the manufacturer’s instructions (Promega, Madison, WI, USA) using 30 *μ*g protein.

### ROS (H_2_O_2_) analysis

ROS assays were conducted using an OxiSelect Hydrogen Peroxide Assay in accordance with the manufacturer’s instructions (Cell Biolabs Inc.). A serial dilution of H_2_O_2_ (0–100 *μ*M) was prepared to generate a standard curve, while U87 and A172 cells (1×10^7^) were plated, exposed to 5 *μ*M ACA or vehicle (DMSO) for 24 h and then sonicated. Subsequently, standards and samples were mixed with an aqueous working reagent (per the manufacturer’s instructions), incubated on a shaker for 30 min at room temperature and absorbances were read using an ELx808 Absorbance Microplate Reader (BioTek, Winooski, VT, USA) at 595 nm.

### Cytokine array assays

U87 and A172 cells were plated overnight, treated with 5 *μ*M ACA or vehicle (DMSO) for 2 h and lysed with CelLytic M Cell Lysis reagent. The protein concentrations were determined using the Bradford method. Cytokine array assays were conducted using a Human 4-Plex Cytokine Array according to the manufacturer’s instructions (Quansys Biosciences, Logan, UT, USA). A serial dilution was performed with a positive control standard antigen in order to generate variable cytokine expression. Diluted standards and 20 *μ*g of protein lysis extract from the treated and vehicle controls were added to wells containing 4-plex arrays (IL-1α, IL-4, IL-6, TNFα and a reference spot) and incubated on a plate shaker for 1 h at room temperature. The wells were then washed 3 times with washing buffer, a detection mix was added and the wells were again incubated on a plate shaker for 1 hour at room temperature. Next, the wells were washed 3 times, streptavidin-horseradish peroxidase (HRP) was added for 15 min, followed by 6 washes, and a substrate for the detection of the cytokines was added. Subsequently, the multiplex plate arrays were imaged using a Q-View imager (Quansys Biosciences).

## Results

### ACA inhibits glioblastoma cell proliferation and migration

**Figure 1:**
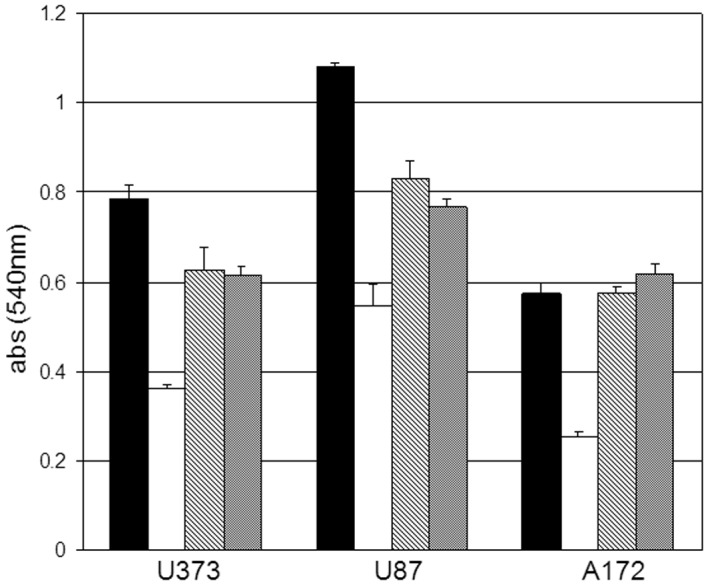
Dose response of ACA on glio blastoma cells. Glioblastoma cell proliferation decreased in response to increasing concentrations of ACA. Solid black bars, vehicle-treated (DMSO) control cells; white bars, 10 *μ*M ACA; hatched bars, 5 *μ*M ACA; gray bars, 2 *μ*M ACA. Data shown are representative of 3 independent experiments (means ± SE) performed in duplicate showing similar results. Abs, absorbance; ACA, 1′-acetoxychavicol acetate.

**Figure 2:**
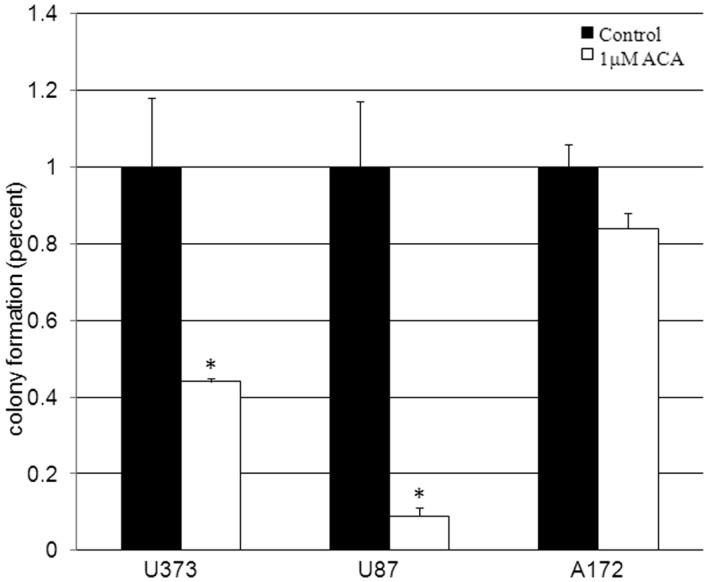
Clonogenic survival of glioblastoma cells treated with ACA. ACA inhibited the clonogenic survival of glioblastoma cells. Black bars, vehicle (DMSO)-treated control cells; white bars, U373, U87 and A172 cells treated with 1 *μ*M ACA. Data shown are representative of at least 3 independent experiments performed in duplicate (means ± SE) with comparable results ^*^P<0.05 compared to vehicle-treated control cells. ACA, 1′-acetoxychavicol acetate.

In the present study, the effects of the xanthine oxidase inhibitor, ACA, on glioblastoma cells were initially evaluated with dose-response experiments, which displayed an overall decrease in cell proliferation in response to increasing concentrations of ACA 48 h post-exposure. Glioblastoma cells treated with 10 *μ*M ACA displayed the most significant inhibition on cell proliferation compared to the vehicle-treated control cells (Fig. 1). U373 and U87 cell proliferation was also impaired by exposure to 5 *μ*M and 2 *μ*M ACA (Fig. 1) in contrast to the A172 cells treated with the same concentrations of ACA. To further assess the cytotoxic effects of ACA on glioblastoma cells clonogenic survival assays were conducted. Clonogenic survival assays utilize low cell plating densities (<500 cells) to evaluate the ability of single cells to form colonies, making it a sensitive assay that assesses cellular reproductive capacity and mimics the clonal expansion behavior of human cancers. Clonogenic survival data revealed statistically significant (P<0.05) decreases of 56 and 91% in the cellular reproductive capacity of the U373 and U87 cells, respectively, when treated with 1 *μ*M ACA (Fig. 2) compared with the vehicle-treated control cells. These data are consistent with previous studies on leukemia (12) and breast cancer (14) cells that also demonstrated the anti-proliferative effects of ACA on these human cancers.

**Figure 3:**
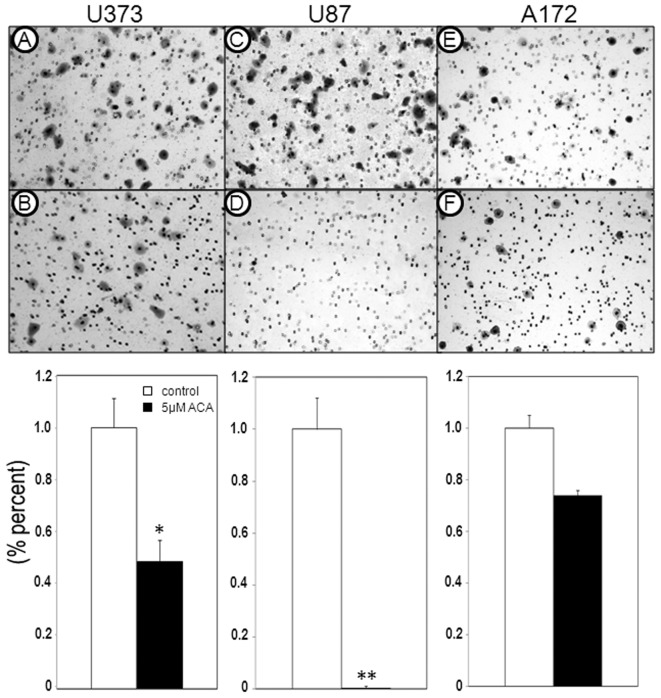
Motility of glioblastoma cells treated with the antioxidant ACA. (A–F) Motility was determined using polycarbonate membrane inserts. (A, C and E) Vehicle (DMSO)-treated control cells. (B, D and F) U373, U87, and A172 cells treated with 5 *μ*M ACA. The antioxidant ACA impairs glioblastoma cell motility. The experiment shown is representative of 3 independent experiments performed in duplicate (means ± SE) that displayed similar results (^*^P<0.05, ^**^P<0.01). ACA, 1′-acetoxychavicol acetate.

**Figure 4:**
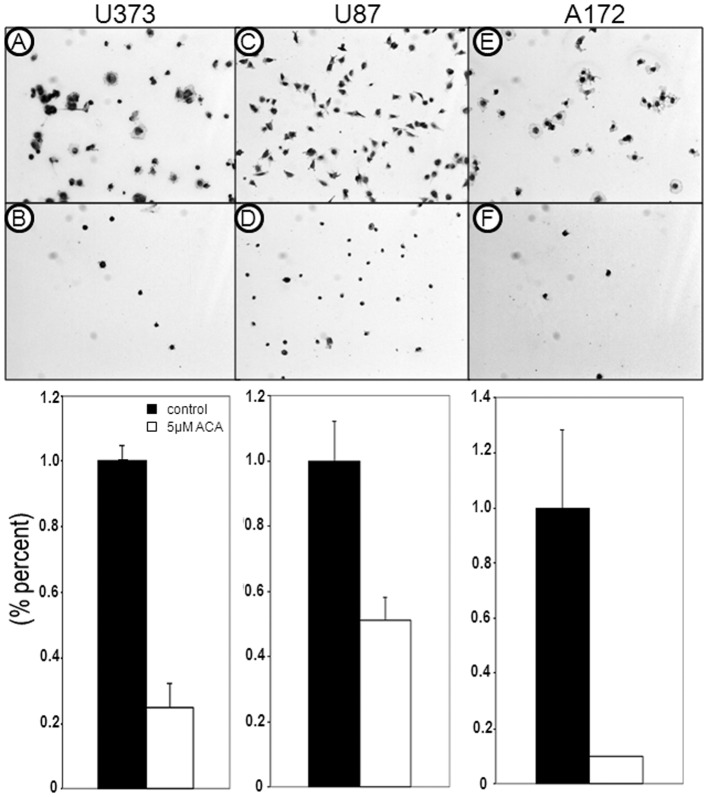
ACA impairs adhesive properties of glioblastomas. (A, C and E) Vehicle (DMSO)-treated control cells; (B, D and F) U373, U87 and A172 cells treated with 5 *μ*M ACA. The experiment shown is representative of 3 independent experiments performed in duplicate (mean ± SE) that displayed similar results. ACA, 1′-acetoxychavicol acetate.

To date, little is known regarding the efficacy of xanthine oxidase inhibitors on cell migration, particularly tumor cell migration. Therefore, in addition to examining the effects of ACA on glioblastoma cell proliferation, the ability of ACA to antagonize glioblastoma cell migration was also assessed. Migration assays revealed a reduction in the migratory ability of the glioblastoma cells following 5 *μ*M ACA exposure (Fig. 3); the most significant effect was observed in the U87 cells, which showed a 97% reduction in cell migration (P<0.01), followed by the U373 cells, which displayed a 51% reduction in cell migration (P<0.05) compared with the vehicle-treated control cells. In addition, ACA also caused a reduction in glioblastoma cell adhesion (Fig. 4), paralleling the observed decrease in glioblastoma cell migration post-ACA treatment.

### Caspase 3-activated cell death

**Figure 5:**
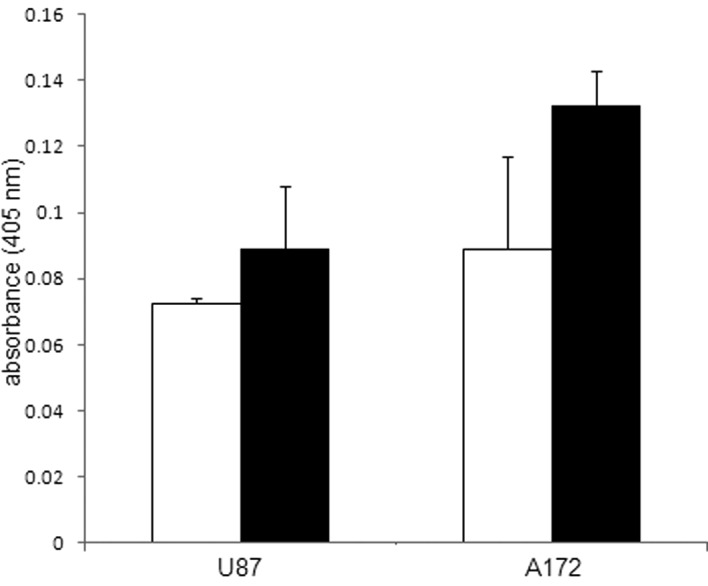
Assessment of caspase 3 activity in U87 and A172 glioblastoma cells in response to 2 *μ*M ACA. Caspase 3 protein activity was measured 24 h post-exposure to ACA (white bars, vehicle control; black bars, ACA). Caspase 3 activity was enhanced in U87 and A172 glioblastoma cells in response to 2 *μ*M ACA. Data is displayed as the mean ± SE of 3 separate independent experiments. ACA, 1′-acetoxychavicol acetate.

**Figure 6:**
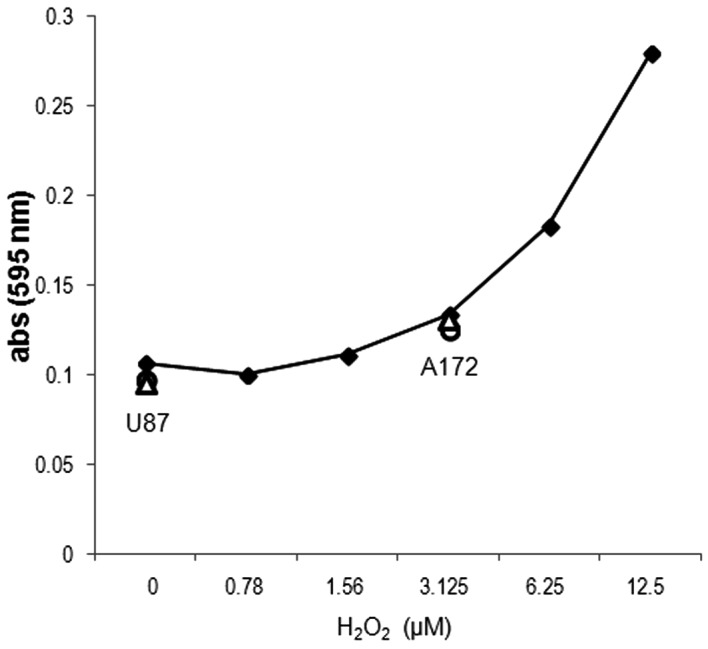
Evaluation of ROS levels as determined by measuring H_2_O_2_ concentration in U87 and A172 cells treated with 5 *μ*M ACA. ACA had no effect on ROS levels. ♦, H_2_O_2_ standard serial dilution; ○, vehicle control treated cells; Δ, cell treated with 5 *μ*M ACA. Abs, absorbance; ROS, reactive oxygen species; ACA, 1′-acetoxychavicol acetate.

The cytotoxic and apoptotic-inducing ability of *Alpinia galanga* rhizome extract and ACA have been demonstrated in several types of human cancers (12–19). Therefore, by evaluating caspase 3 activity, the present study examined whether the observed inhibitory effects of ACA on glioblastoma cell production were a consequence of apoptotic cell death. Caspase 3, an effector (executioner) caspase of apoptotic cell death, was measured in the glioblastoma cells treated with 2 *μ*M ACA. Increases in caspase 3 activity of 23 and 49% were observed in the U87 and A172 glioblastoma cells, respectively, compared with the vehicle-treated control cells (Fig. 5). This data provides evidence that indicates that glioblastoma cells undergo apoptosis in response to ACA exposure and is consistent with a study by Moffatt *et al*, which demonstrated that ACA promoted apoptotic cell death in Ehrlich ascites tumor cells via caspase 3 activation (15). In addition to assessing caspase 3 involvement during ACA-induced glioblastoma cell death, ROS, which are known to influence tumor cell viability were also studied in the ACA-treated glioblastoma cells. An evaluation of the ROS levels was performed by measuring the H_2_O_2_ concentration; however, no notable difference was observed between glioblastoma cells exposed to 5 *μ*M ACA and the vehicle-treated control cells (Fig. 6).

### ACA-induced cytokine expression

**Figure 7:**
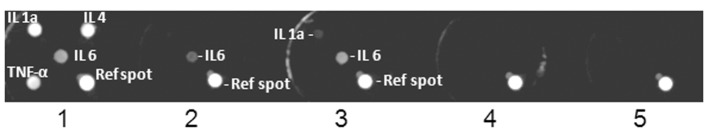
Cytokine array analysis. Induction of cytokine expression (IL-6 and IL-1α) by ACA. (1) Cytokine antigen standard; (2) U87 vehicle-treated control cells; (3) U87 cells treated with 2 *μ*M ACA; (4) A172 vehicle-treated control cells; (5) A172 cells treated with 2*μ*M ACA. Experiment shown is representative of 3 independent experiments performed in duplicate that displayed similar results. ACA, acetoxychavicol acetate.

Cytokines are well-recognized as molecules that contribute to the development, progression and maintenance of several types of human cancers as a consequence of pleiotropic signaling mechanisms that promote the survival of tumor cells (20,21). Recent studies have demonstrated that ROS promote cytokine production (22) and provide mechanistic support for cytokine activity. The present study therefore evaluated the effect of ACA on cytokine expression in glioblastomas (Fig. 7), which are known to express IL-6 at high levels compared with normal brain tissue, and have been shown to play a prominent role in glioblastoma cell survival and migration (23–26). A cytokine expression array analysis revealed that the treatment with 2 *μ*M ACA invoked the increased expression of IL-6 and IL-1α in the U87 cells compared with the vehicle-treated controls, while cytokines were not detected in the A172 cells (Fig. 7).

## Discussion

Antioxidants are often studied for their properties as chemopreventive agents. However, in the present study, the utility of the antioxidant, ACA, was examined for its anti-tumorigenic properties on glioblastomas. Glioblastomas, like normal brain tissue, have low antioxidant activity. This is supported by several studies that have revealed depleted levels of ROS detoxification enzymes (gluthathione peroxidase and superoxide dismutase) in clinical glioblastomas examined from tumor patient explants (27–29). Additionally, glioblastomas have been shown to increase the expression levels of the ROS generator, xanthine oxidase. Together, these studies suggest that ROS, which have been shown to have pro-tumorigenic effects, contribute to the survival, maintenance and progression of glioblastomas.

The present study demonstrated that ACA antagonizes glioblastoma cell proliferation as a consequence of promoting caspase 3-induced apoptotic cell death. These findings parallel results previously shown in myeloma (13), leukemia (12) and breast cancer studies (14), which also demonstrated that ACA invoked an apoptotic cell death response as a result of increased caspase 3 activity. In contrast to the well-characterized inhibitory effects of ACA on tumor cell proliferation, few studies have been conducted on the efficacy of ACA to prevent tumor cell metastatic invasion. The present study revealed that ACA impeded glioblastoma cell migration by impairing the adhesive properties of the cells, suggesting that ACA diminishes the invasive capacity and growth of glioblastomas at secondary tumor sites in brain tissue by blocking cell motility. Consistent with these data, In *et al*(30) and Ichikawa *et al*(17) also demonstrated that ACA inhibited the migratory invasiveness of human oral carcinomas in mouse xenografts and lung cancer cells, respectively.

Although the present study established that ACA is an effective antagonist of glioblastoma cell proliferation and migration, it is not likely to be a consequence of the ACA antioxidant functions, as evidenced by ROS experimental data, which revealed no change in the H_2_O_2_ concentration between the control and glioblastoma cells treated with ACA. Therefore the study also examined the mechanistic effects of ACA on the cytokines, pro-inflammatory and tumorigenic activator molecules of the JAK-STAT mitogenic pathway, which have been shown to be upregulated by ROS (22) and act as pro-survival molecules in brain tumors. Recent studies in glioblastomas have demonstrated that the cytokine IL-6 plays a role in enhancing glioblastoma cell survival and migratory invasion (23–26), while studies in neuroblastomas demonstrated that IL-6 and IL-1α promote cell survival by acting as protectors of these nervous system-derived cancers (31). In contrast to the expected effects of ACA on cytokine levels, in the present study, an increased expression of IL-6 and IL-1α was observed in glioblastoma cells treated with ACA. This provided evidence that indicated that glioblastoma cells elicit a compensatory pro-survival response in addition to a pro-apoptotic ACA-induced caspase 3 response.

To the best of our knowledge, this is the first study that provides an insight into the function and versatility of ACA to promote tumor cell death by circumventing the pro-survival signaling mechanisms that are likely to contribute to the therapeutic resistance of human cancers, in general and in glioblastomas in particular. The novelty of ACA to overcome the involvement of molecular signaling molecules associated with perpetuating the maintenance and progression of human cancers make it an attractive single and combinatorial drug agent for use in continued experimental and clinical studies for the treatment of this disease.
